# Editorial

**Published:** 1898-12

**Authors:** 


### Editorial.

The Red Cross people seem to have furnished the low comedy of our recent opera bouffe affair with Spain. Their conduct has been marked by a tremendous display of zeal without wisdom.

Richard Harding Davis says that in the American occupation of Ponce the local fire companies paraded and ran over three of their own men, which gave the Red Cross people a grand chance to appear on the scene, each man wearing four red crosses, to carry away the wounded. "This created some confusion, as the firemen preferred to walk, but the Red Cross people were adamant and bore them off on stretchers whether they would or no." The High Priestess of the Rosicrucians, Miss Clara Barton, in furnishing a testimonial of a quack "nervine," has given evidence of a venality which puts her quite on a plane with rural Congressmen and Paine's Celery Compound clergymen.

Following this testimonial is her endorsement of Electropoise, an instrument which has been defined as consisting of a cord with a cylinder at one end and a fool at the other. This is her letter:

CONSTANTINOPLE, Feb. 21, 1896.

DEAR SIR: When in London the other day I received two packets from the United States Embassy, each containing an Electropoise; to-day I received your kind letter. Please allow me to thank you heartily and gratefully for the splendid little machines. As you remember, I am not an entire stranger to the virtues of the Electropoise, and I will take great pleasure in passing your offering to afflicted humanity. Very sincerely yours,

CLARA BARTON,
President Red Cross Armenian Relief Expedition.

As the "splendid little machine" "when conscientiously used will cure catarrh, neuralgia, hay fever, insomnia, nervous and women's troubles," it is presumed that the Armenians afflicted with these ailments will receive the relief for which they have so long waited in vain. We would venture the suggestion that she might strike nearer the root of their troubles by tying one of the splendid little machines to the kœniglische, kaiserlische leg of the Sick Man of the East himself. What a beautiful picture would this be! Miss Barton rushes into a hovel in Erzeroum with a dollar-size bottle of Dr. Green's Nervura in one hand and dragging a splendid little machine by the other. It is but the work of a moment to attach the magic cord to the wasted limb of the sufferer and to press the life-giving fluid to his parched and withered lips, then see the sunken cheek mantle with the rushing red tide and the feeble limbs gain strength until the sufferer rises to his feet completely restored to strength and ready to chant a song of praise to his rescuer and to hurl renewed defiance in the teeth of the Mussulmans. Like results might be secured from increasing quantities of "tender bosom" and corn bread, but these banalities are inconsistent with the high and noble aims of the Red Cross leader.

It is not enough for Miss Barton to fly in the face of the just and deeply rooted prejudices of the medical profession, which she should regard as her closest ally, by publicly endorsing the cheapest quackeries; she must needs use her office to further their commercial interest.

As the Red Cross Society is sustained by public charity, the public becomes backers of the quacks, and certainly there are other and more laudable means of contributing to the aid of the afflicted than by giving alms through the medium of an emissary of charlatanry. It seems that the Red Cross people have outlived their usefulness.

---

Dr. T. H. Huzza, a well-known physician of this city, died in New York Hospital from appendicitis, December 9th. He was thirty-five years of age and unmarried.

---

Vol. XXVII. ends with this number. We begin the twenty-eighth year of continuous publication with some pride. We have withstood the heavy strain of the last four years successfully. We have made new friends and have retained the patronage and good will of the old. Our advertisers seem pleased with us and we have encouraging words from our subscribers. In this day of mushroom medical journalism it is with pardonable pride that we call attention to these things. We base our hopes for the future on the past and have no fears.

